# A randomised clinical trial to evaluate the effect of a 67 % sodium bicarbonate-containing dentifrice on 0.2 % chlorhexidine digluconate mouthwash tooth staining

**DOI:** 10.1186/s12903-016-0271-3

**Published:** 2016-08-25

**Authors:** Ivy Akwagyiram, Andrew Butler, Robert Maclure, Patrick Colgan, Nicole Yan, Mary Lynn Bosma

**Affiliations:** 1 GSK Consumer Healthcare, St Georges Avenue, Weybridge, Surrey KT13 0DE UK; 2 Intertek Clinical Research Services: Site 1, Unit 4 Enterprise House, Manchester Science Park, Lloyd Street North, Manchester, M15 6SE UK; 3 Intertek Clinical Research Services: Site 2, 32 High Street, Maldon, Essex CM9 5PN UK

**Keywords:** Chlorhexidine mouthwash, Sodium bicarbonate, Dentifrice, Stain

## Abstract

**Background:**

Gingivitis can develop as a reaction to dental plaque. It can be limited by curtailing plaque build-up through actions including tooth brushing and the use of medicinal mouthwashes, such as those containing chlorhexidine digluconate (CHX), that can reach parts of the mouth that may be missed when brushing. This study aimed to compare dental stain control of twice-daily brushing with a sodium fluoride (NaF) dentifrice containing 67 % sodium bicarbonate (NaHCO_3_) or a commercially available NaF silica dentifrice without NaHCO_3,_ while using a mouthwash containing 0.2 % CHX.

**Methods:**

This was a 6-week, randomised, two-site, examiner-blind, parallel-group study in healthy subjects with at least ‘mild’ stain levels on the facial surfaces of ≥4 teeth and ≥15 bleeding sites. Assessment was via modified Lobene Stain Index (MLSI), the score being the mean of stain intensity multiplied by area (MLSI [IxA]).

**Results:**

One hundred and fifty of 160 randomised subjects completed the study. There were no significant differences in Overall (facial and lingual) MLSI (IxA) scores between dentifrices. The Facial MLSI (IxA) was statistically significant at 6 weeks, favouring the 67 % NaHCO_3_ dentifrice (*p* = 0.0404). Post-hoc analysis, conducted due to a significant site interaction, found significant differences for all MLSI scores in favour of the 67 % NaHCO_3_ dentifrice at Site 1 (both weeks) but not Site 2.

**Conclusions:**

No overall significant differences were found between a 67 and 0 % NaHCO_3_ dentifrice in controlling CHX stain; a significant difference on facial surfaces suggests advantage of the former on more accessible surfaces.

**Trial registration:**

This study was registered at ClinicalTrials.gov (NCT01962493) on 10 October 2013 and was funded by GSK Consumer Healthcare.

## Background

Gingivitis, a common problem that in some can lead to periodontitis, can develop as a reaction to dental plaque [1]. The occurrence of gingivitis can be limited by curtailing plaque build-up through actions including tooth brushing and the use of medicinal mouthwashes that can reach parts of the mouth that may be missed when brushing [2, 3].

Chlorhexidine digluconate (CHX), used in dentistry for around 40 years [3], has antibacterial properties against both gram-positive and gram-negative species [1, 4] including those associated with periodontal disease [5]. Following use of CHX-containing mouthwash, about a third of the active ingredient remains on the teeth, pellicle, oral mucosa, tongue and in salivary proteins [3, 6], providing sustained antibacterial properties for 8–12 h [6]. These actions, especially when combined with tooth brushing, can lead to break up of existing plaque, reduction of plaque re-growth and inhibition of the development of gingivitis [7–10].

CHX mouthwashes may be recommended by dental professionals for use over a period of a few weeks or months in those for whom gingivitis is problematic [10]. However, CHX is associated with significantly increased levels of staining compared to non-CHX mouthwashes [10, 11], which may lead to reluctance to use a CHX mouthwash as prescribed. Adding a toothpaste with good stain control qualities to this oral healthcare regimen could make the use of a CHX mouthwash more acceptable to those concerned about staining. As staining is associated with the presence of plaque [12], measures to try and prevent CHX staining could include use of dentifrices with plaque-limiting ingredients. One such ingredient is sodium bicarbonate (NaHCO_3_), which works by enhancing physical removal of plaque biofilm [13]. An added advantage of NaHCO_3_ is that it has very low abrasivity [14], thus lowering potential damage found with some stain-limiting ingredients with high abrasivity [15].

To investigate the stain control properties of NaHCO_3_, this study compared twice daily brushing with a sodium fluoride (NaF) dentifrice containing 67 % NaHCO_3_ versus a commercially available NaF silica dentifrice without NaHCO_3_ during 6 weeks use of a mouthwash containing 0.2 % CHX.

## Methods

This 6-week, randomised, single centre (Intertek Clinical Research Services, UK), two site (Site 1: Manchester, UK; Site 2: Maldon, UK) examiner-blind, parallel-group study under the same Principal Investigator compared the stain control effect of twice daily brushing with a 67 % NaHCO_3_-containing dentifrice versus a standard dentifrice when both were used in combination with a 0.2 % CHX mouthwash. This study was reviewed by the National Research Ethics Service Committee East Midlands, Northampton. There were two protocol amendments, both for purely administrative reasons, of which the review committee was informed. This study is in ClinicalTrials.gov (NCT01962493) with results published on the GSK Clinical Study Register website [16].

During screening, subjects gave their written informed consent to participate in the study in accordance with the Declaration of Helsinki [17]. Demographics, medical history and concomitant medications were recorded, followed by an oral examination (which included an oral soft tissue [OST] examination), gross stain assessment and a gingival bleeding assessment (see below) to confirm study suitability. Subjects were 18–64 years of age, in good general and oral health (excluding gingivitis) with a minimum of 11 of 12 permanent gradable anterior teeth and, as a means to assess the presence of gingivitis, a total of at least 15 bleeding sites as measured by the Saxton & Van der Ouderra Bleeding Index (BI, a modification of the Gingival Index) [18] where 0 = no bleeding after 30 s, 1 = bleeding upon probing after 30 s, 2 = immediate bleeding observed; the score was converted to bleeding (score of 1 or 2) or no bleeding (score of 0) to determine the number of bleeding sites in the mouth.

**Fig. 1:**
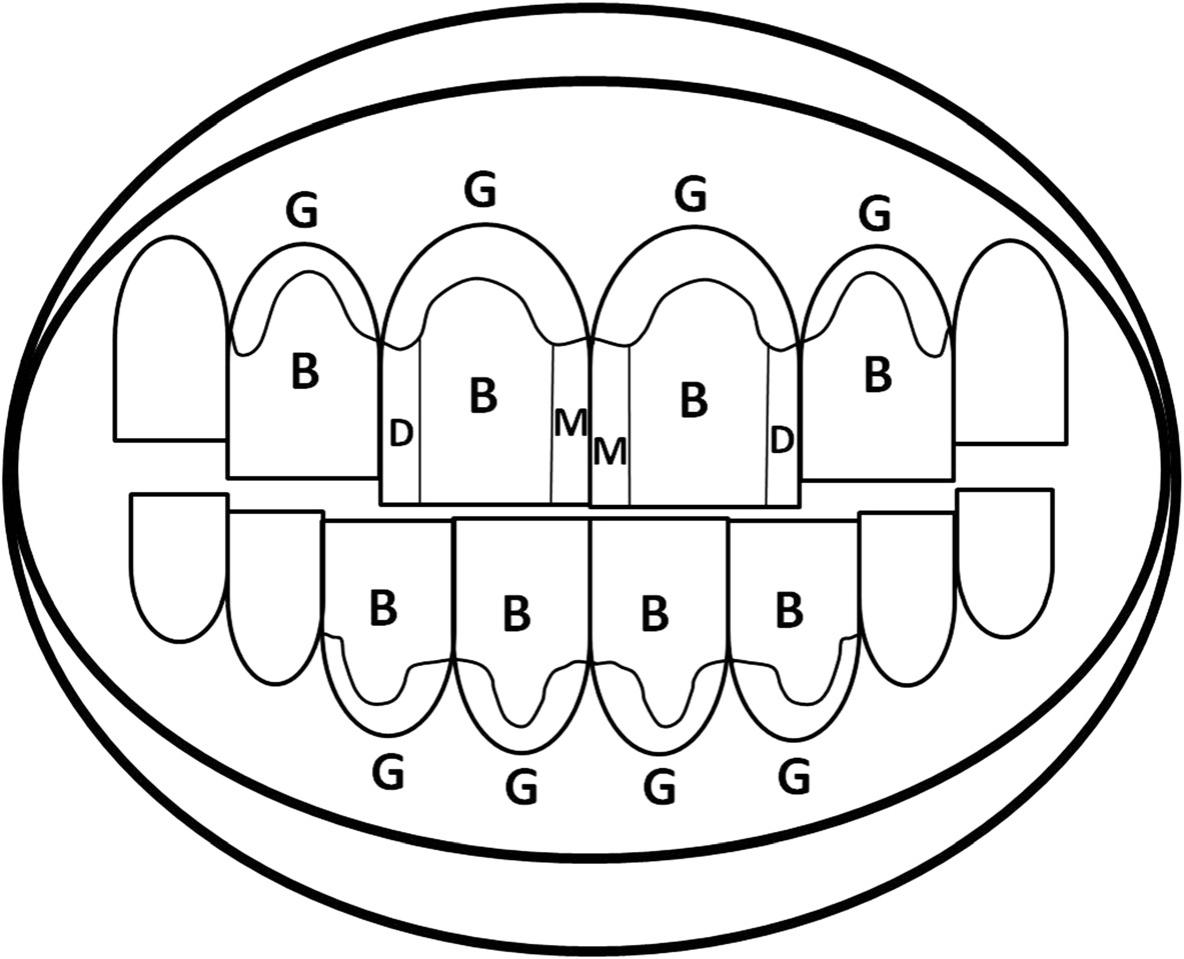
Diagram of MacPherson tooth areas with MLSI grading sites on the two central upper incisors. G = Gingival; B = Body; M = Mesial; D = Distal

Following screening, eligible candidates were provided with a standard washout dentifrice (Control dentifrice, see below) for use at home for one timed minute twice daily, applied in a strip to cover the head of a supplied toothbrush (Aquafresh Clean Control Medium toothbrush, GSK Consumer Healthcare [GSKCH], Weybridge, UK, to be used throughout the study) until the baseline visit (up to 14 days after screening). At this visit subjects underwent a full OST exam and a stain assessment using the modified Lobene Stain Index (MLSI) on MacPherson sites (see description below and Fig. 1). Intra-oral digital images were taken of the relevant facial and lingual surfaces of the anterior teeth to provide a record of tooth stain before and after product use, these were not used for stain rating. A dental prophylaxis and floss was carried out on the anterior teeth used to assess the stain to clean and remove all stain to ensure they were free of all plaque and supra- and sub-gingival calculus both visually and by tactile assessment. Hence, all subjects had an MLSI score of 0 prior to study treatment use. Throughout the study the use of other oral care products including other mouthwashes, antimicrobial lozenges, chewing gums or floss (except for impacted food removal) was not allowed.

A randomisation schedule generated by the Biostatistics and Data Management Department of GSKCH stratified subjects based on site, baseline MLSI score (low Facial MLSI <45, high Facial MLSI ≥45) and smoking status. All subjects received mint flavour, alcohol free Corsodyl® 0.2 % mouthwash containing 0.2 % CHX (GSKCH, Weybridge, UK). Subjects were allocated to either a NaHCO_3_ dentifrice: Corsodyl® Daily Gum and Toothpaste containing 67 % NaHCO_3_ and 1400 ppm fluoride as NaF (relative dentin abrasivity [RDA] ∼47.6) (GSKCH, Weybridge, UK) or a Control dentifrice without NaHCO_3_: Aquafresh® Fresh & Minty Toothpaste containing 1450 ppm fluoride as NaF (RDA ∼93.0) (GSKCH, Weybridge, UK). Study dentifrices were supplied in commercial packaging then over-wrapped to blind study staff and subjects to treatment assignment. CHX mouthwash was supplied in its commercial packaging.

Subjects were instructed to apply a strip of dentifrice to cover the toothbrush head and brush in their usual manner for one timed minute then thoroughly rinse their mouth with water. Five timed minutes after brushing, subjects rinsed for one timed minute with 10 mLs of 0.2 % CHX mouthwash. To ensure treatment compliance, subjects’ first use of their allocated products was carried out under supervision at the site. Subjects then used their assigned product at home twice daily for 6 weeks, with use reported on a supplied diary card that was checked at all study visits. At Weeks 3 and 6 ± 2 days post baseline, subjects returned to the site and underwent a full OST examination followed by stain assessments using the MLSI index.

### Objectives

The primary objective was to determine whether brushing with a 67 % NaHCO_3_ dentifrice produced a greater level of stain control than brushing with a non-NaHCO_3_ dentifrice following 6 weeks usage, as indicated by Overall (total facial and lingual) MLSI stain Intensity multiplied by Area (IxA) scores. Secondary objectives were to compare Overall MLSI treatment differences following 3 weeks of usage; to compare differences between treatments on stain-control levels after 3 and 6 weeks of treatment, as indicated by the individual MLSI (IxA) scores for each region (Overall Interproximal, Overall Facial, Overall Gingival and Interproximal), and to monitor oral adverse events (AEs) using OST examination.

### Procedures and assessments

#### Gross stain assessment

An examination to ascertain oral health and gross level of stain was performed. Subjects needed to have a sufficient level of stain at screening to be eligible. Stain levels on the facial surfaces of the six maxillary and six mandibular anterior teeth needed to be at least ‘mild’ (i.e. mild, moderate or severe) and present on a minimum of four teeth out of the 12 maxillary and mandibular teeth evaluated.

#### MLSI using MacPherson’s sites [19, 20]

Stain was assessed on air-dried teeth by a single qualified examiner after the subject had brushed the teeth to be assessed with water for 10 s to remove any loose debris. Assessment was made of the area and intensity of dental stain on the facial surfaces of the maxillary and mandibular anterior teeth and lingual surfaces of the mandibular anterior teeth, surfaces that cosmetically are the most apparent with respect to stain accumulation. Each surface was divided into four regions (see Fig. 1).

- Gingival (G) = a crescent-shaped band about 2 mm wide running parallel to the gingival margin; the limit of the G region is toward the incisal edge, marked by the end of the interdental papilla
- Body (B) = the central area of the buccal/lingual aspect, between G and D/M sites, extending to the incisal edge
- Mesial (M) = the visible area of the tooth facing *toward* the central midline, ending at the interdental papilla (G site start), bordered by the B area
- Distal (D) = the visible area of the tooth facing *away from* the central midline, ending at the interdental papilla (G site start), bordered by the B area

The MLSI is derived from the products of the Intensity and Area scores (IxA scores) of all regions.

#### Stain intensity

The intensity of stain was scored separately for each region using the following criteria:

- 0 = No stain
- 1 = Light stain
- 2 = Moderate stain
- 3 = Heavy stain

#### Stain area

The area of stain was scored separately for each region using the following criteria:

- 0 = No stain
- 1 = Stain up to 1/3 of the area affected
- 2 = Stain between 1/3 and 2/3 of the area affected
- 3 = Stain more than 2/3 of area affected

The MLSI (IxA) scores were calculated at the site level first and then averaged over the whole regions of interest (Overall, Overall Interproximal, Overall Facial, Overall Gingival and Interproximal), as detailed in Table 1.

**Table 1:** Relevant tooth site for calculation of MLSI score

Variable	Surfaces	Region	Summary (Average over)
Overall MLSI	Max-F, Man-F, Man-L (18)	GBMD (4)	18 × 4 = 72 sites
Overall Facial MLSI	Max-F, Man-F (12)	GBMD (4)	12 × 4 = 48 sites
Overall Interproximal MLSI	Max-F, Man-F, Man-L (18)	MD (2)	18 × 2 = 36 sites
Overall Gingival and Interproximal MLSI	Max-F, Man-F, Man-L (18)	GMD (3)	18 × 3 = 54 sites

*Max-F* maxillary-facial, *Man-F* mandibular-facial, *Man-L* mandibular-lingual, *G* gingival, *B* body, *M* mesial and *D* distal

#### OST assessment

The OST examination was accomplished by direct observation and palpation with retraction aids as appropriate. Any post-treatment soft tissue abnormality observed by the examiner or reported by the subject was recorded as an adverse event.

All adverse events, treatment-related or not, were also recorded.

### Statistical analysis

#### Sample size

It was planned to screen sufficient number of subjects to randomise a maximum of 160 (80 to each treatment group), ensuring at least 70 evaluable subjects per group completed the Week 6 assessment, this being the number calculated to provide 90 % power to detect a difference between treatment groups of 0.364 in the MLSI (IxA) at Week 6, assuming a standard deviation of 0.66 [21], using a two sample *t*-test with a 0.05 two-sided significance level.

The primary population for assessment of efficacy was the intent-to-treat (ITT) population, defined as those who received study treatment and had at least one post-baseline efficacy measurement. Analysis of Covariance (ANCOVA) was used to analyse the MLSI (IxA) at Weeks 3 and 6. The model included treatment, site and smoking status as factors and the relevant baseline MLSI (IxA) score as a covariate. A factor for treatment by study site interaction was included and retained in the model as it was significant at the 10 % level. Since the treatment by site interaction was significant, a post-hoc analysis by study site was carried out with factors for treatment and smoking status and baseline as a covariate. Residuals from the model showed departures from normality and a square root transformation improved the distribution and was applied to the data. Resulting means and confidence intervals (CIs) were back transformed [22].

#### Assessment of examiner

Weighted Kappa coefficients, along with 95 % CIs, were calculated to assess the intra-examiner repeatability for MLSI (IxA) assessments at each visit. Repeatability was deemed to represent excellent replication if the kappa coefficient was greater than 0.75, fair to good replication if the value was between 0.4 and 0.75 inclusive and poor replication if the value was below 0.4.

## Results

### Baseline summary

In total, 314 subjects were screened and 160 were randomised (99 at Site 1, 61 at Site 2), of whom 150 completed the study. All 160 subjects were treated so were included in the Safety population. Their mean age was 39.6 (range 18–64), they were mainly female (72.5 %), white (78.1 %) and non-smokers (77.5 %). Most subjects had a facial MLSI (IxA) score at baseline of <45 (90.0 %) with a mean baseline BI score of 0.5, comparable for each group (Table 2).

**Table 2:** Summary of the demographics and baseline characteristics for the Safety population (*n* = 160)

		67 % NaHCO_3_ dentifrice + mouthwash *N* = 78	Control dentifrice + mouthwash *N* = 82
Male n (%)		22 (28.2 %)	22 (26.8 %)
Female n (%)		56 (71.8 %)	60 (73.2 %)
Mean age (±SD)		39.8 (11.08)	39.5 (10.87)
Age range		19–64	18–63
Ethnicity n (%)	White	56 (71.8)	69 (84.1)
	Black	9 (11.5)	5 (6.1)
	Asian	9 (11.5)	2 (2.4)
	Multiple	4 (5.1)	6 (7.3)
Smoker n (%)	Yes	18 (23.1)	18 (22.0)
	No	60 (76.9)	64 (78.0)
Total facial MLSI (IxA) score n (%)	Low (<45)	72 (92.3)	72 (87.8)
	High (≥45)	6 (7.7)	10 (12.2)
Mean Bleeding Index (±SD)		0.5 (0.50)	0.5 (0.50)
Mean Overall MLSI (IxA) (±SD)^a^		0.78 (0.710)	0.74 (0.610)
Site n (%)	1	49 (62.8)	50 (61.0)
	2	29 (37.2)	32 (39.0)

^a^From ITT population (*n* = 75, 79); *n* number, *SD* standard deviation

**Fig. 2:**
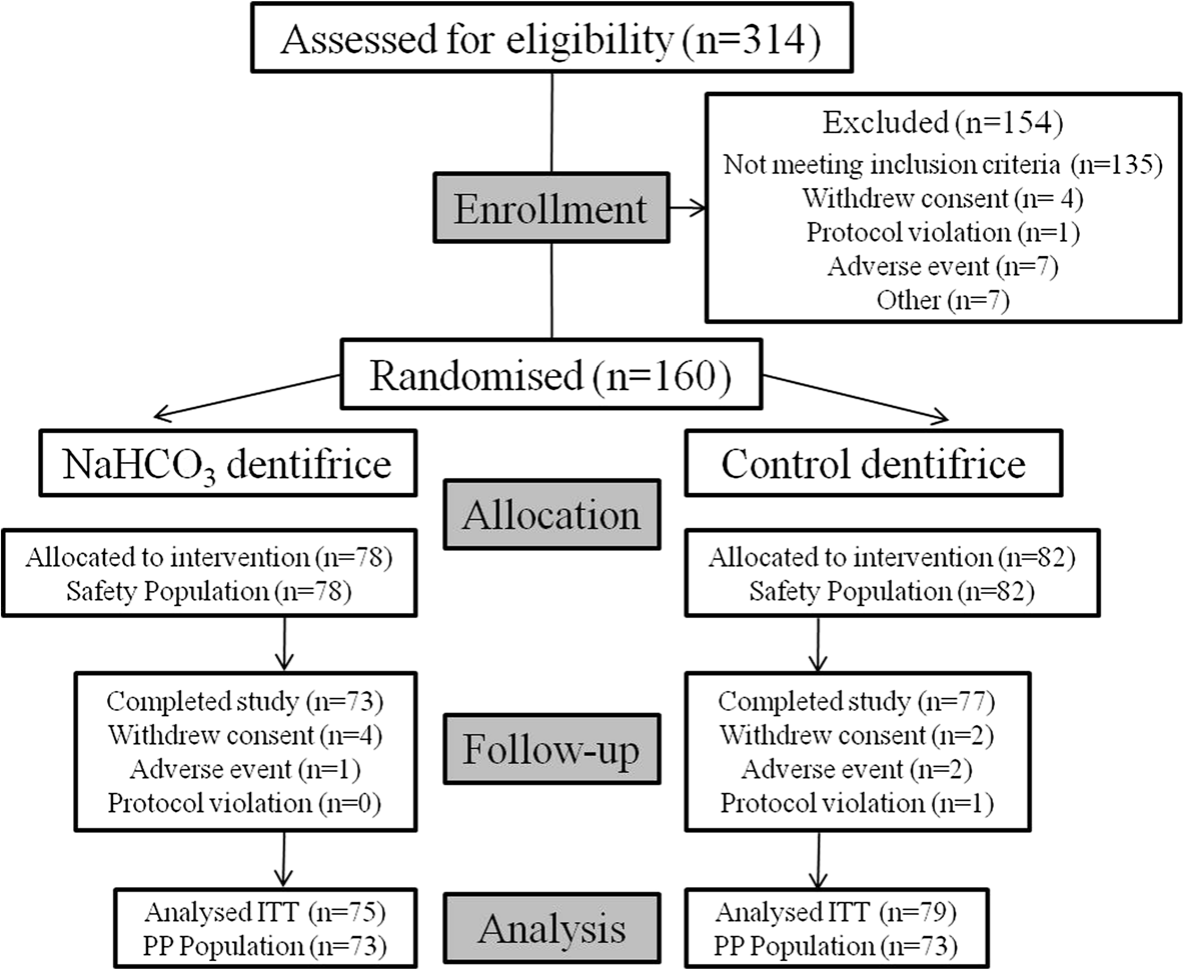
Trial profile. PP = Per Protocol; ITT = Intent-to-treat

Of the 160 subjects, six provided no post-baseline efficacy data leading to an ITT population of 154 (95 at Site 1, 59 at Site 2). Fourteen of the 160 subjects had protocol violations leading to total exclusion and a Per Protocol (PP) population of 146 (Fig. 2). Due to similar numbers between ITT and PP populations (<10 % difference), PP analysis was not performed. The study was carried out between 16 September 2013 and 20 November 2013.

### Efficacy results

**Fig. 3:**
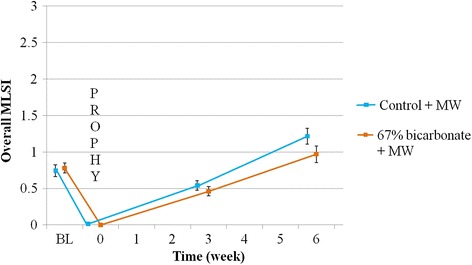
Unadjusted Overall MLSI score by treatment time (± standard error). BL = Baseline; PROPHY = Prophylaxis; MW = Mouthwash; MLSI = modified Lobene Stain Index

For all comparisons, the 67 % NaHCO_3_ dentifrice had numerically lower MLSI scores than the Control dentifrice. However, there was no significant difference between treatments for the primary efficacy variable of Overall MLSI at 6 weeks (−19.9, *P* = 0.1313). The Overall Facial MLSI was significantly different at Week 6, favouring the 67 % NaHCO_3_ dentifrice (−32.2 %, *P* = 0.0404) (Table 3, Fig. 3).

**Table 3:** Summary of treatment differences in MLSI regions by week (ITT population *n* = 154)

Region	Week	Difference (95 % CI)^b^	% Diff^b^	*P*-value
Overall MLSI	3	−0.09 (−0.24, 0.06)	−22.6	0.1552
	6^a^	−0.20 (−0.51, 0.10)	−19.9	0.1313
Overall Facial MLSI	3	−0.09 (−0.20, 0.01)	−39.2	0.0529
	6	−0.23 (−0.49, 0.02)	−32.2	**0.0404**
Overall Interproximal MLSI	3	−0.13 (−0.36, 0.09)	−22.0	0.1873
	6	−0.28 (−0.74, 0.18)	−18.4	0.1713
Overall Gingival and Interproximal MLSI	3	−0.12 (−0.31, 0.07)	−22.8	0.1614
	6	−0.23 (−0.60, 0.15)	−18.2	0.1709

^a^Primary objective; ^b^ *Diff* difference is 67 % NaHCO_3_ dentifrice minus control dentifrice. A negative difference favours the former. Results are based on back transformed data based on square root transformation; bold text indicates a statistically significant value

**Fig. 4:**
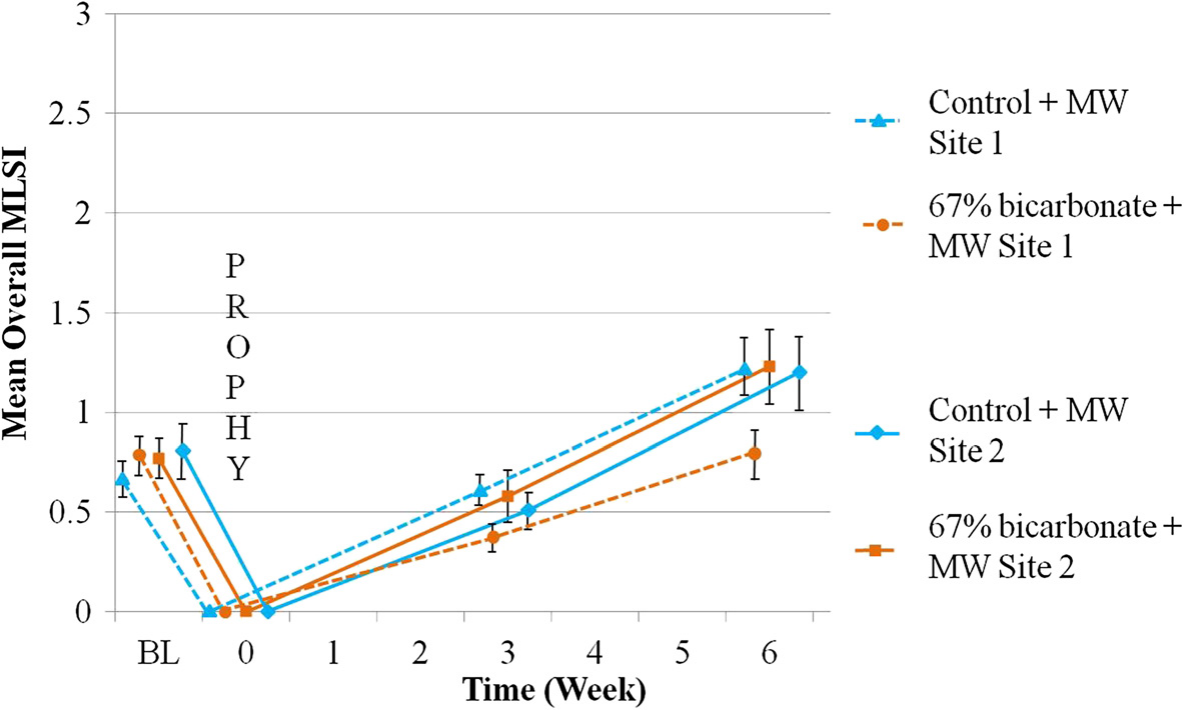
Mean Overall MLSI score by treatment, study site (centre) and time (unadjusted means ± standard error). BL = Baseline; PROPHY = Prophylaxis; MW = Mouthwash; MLSI = modified Lobene Stain Index

There was a significant treatment site interaction; a post-hoc investigation of the data by site showed different treatment effects at each (Table 4; Fig. 4).

**Table 4:** Summary of treatment differences by site (ITT population *n* = 154)

Site	Week	Difference (95 % CI)	% Diff^a^	*P*-value
*Overall MLSI*				
1	3	−0.26 (−0.43, −0.09)	−52.1	**0.0016**
	6	−0.44 (−0.77, −0.10)	−41.2	**0.0072**
2	3	0.08 (−0.16, 0.31)	22.6	0.4621
	6	0.05 (−0.44, 0.54)	5.5	0.8038
*Facial overall MLSI*				
1	3	−0.19 (−0.32, −0.06)	−64.8	**0.0019**
	6	−0.40 (−0.68, −0.12)	−53.0	**0.0032**
2	3	0.00 (−0.17, 0.17)	1.2	0.9757
	6	−0.04 (−0.45, 0.36)	−6.4	0.8105
*Overall interproximal MLSI*				
1	3	−0.36 (−0.62, −0.10)	−49.7	**0.0043**
	6	−0.61 (−1.12, −0.10)	−39.2	**0.0126**
2	3	0.10 (−0.25, 0.45)	19.4	0.5421
	6	0.09 (−0.65, 0.83)	6.0	0.7888
*Overall gingival and interproximal MLSI*				
1	3	−0.31 (−0.53, −0.09)	−51.0	**0.0029**
	6	−0.50 (−0.91, −0.09)	−39.6	**0.0108**
2	3	0.08 (−0.21, 0.38)	19.0	0.5409
	6	0.09 (−0.52, 0.69)	6.9	0.7550

^a^Diff = Difference is 67 % NaHCO_3_ dentifrice minus control dentifrice. A negative difference favours 67 % NaHCO_3_ dentifrice. Results based on back transformed data based on square root transformation; bold text indicates a statistically significant value.

At Site 1, for Overall MLSI (IxA) score at both 3 and 6 weeks there were significant differences of −52.1 % (*P* = 0.0016) and −41.2 % (*P* = 0.0072) in favour of the 67 % NaHCO_3_ dentifrice group. However, no such significant differences were seen at Site 2 for either week. Similarly, all secondary efficacy variables were significantly different for Site 1 and none were significantly different at Site 2.

### Repeatability

The repeatability analysis of the MLSI Index (based on 30 subjects) showed excellent agreement between the first and repeat assessments for both area and intensity. The weighted kappa value for MLSI-Area was 0.91 (95 % CI = 0.89, 0.93) and MLSI-Intensity was 0.92 (95 % CI = 0.90, 0.94).

### Safety results

Overall, 80 subjects (50.0 %) reported at least one AE, with a total of 142 AEs. All of the 31 treatment-related AEs reported by 28 subjects (17.5 %) were oral AEs (Table 5). Three subjects withdrew due to AEs; two in the Control group: one due to dysgeusia and one due to glossodynia, and one subject from the 67 % NaHCO_3_ dentifrice group due to appendicitis and ruptured ovarian cyst. There were three serious AEs reported by two subjects: one had autoimmune pancreatitis, reported pre-treatment, and one had appendicitis and a ruptured ovarian cyst, reported during the study. None of these AEs were treatment related.

**Table 5:** Treatment emergent adverse events (Safety population *N* = 160)

	67 % NaHCO_3_ dentifrice + MW (*N* = 78)		Control dentifrice + MW (*N* = 82)	
	*n (%)*	*nAE*	*n (%)*	*nAE*
All AEs	39 (50.0)	70	41 (50.0)	72
Oral AEs	21 (26.9)	24	33 (40.2)	43
Treatment Related AEs	11 (14.1)	12	17 (20.7)	19
Treatment-related oral AEs				
Dysgeusia	1 (1.3)	1	7 (8.5)	7
Paraesthesia oral	3 (3.8)	3	4 (4.9)	4
Glossodynia	2 (2.6)	2	3 (3.7)	4
Tongue discolouration	4 (5.1)	4	0	0
Ageusia	1 (1.3)	1	2 (2.4)	2
Sensitivity of teeth	1 (1.3)	1	1 (1.2)	1
Hypoaesthesia oral	0	0	1 (1.2)	1

*MW* mouthwash; *n (%)* number (percent) of subjects with at least one AE; *nAE* number of adverse events

## Discussion

Studies have shown that CHX-containing mouthwash is an effective way to reduce gingivitis [7, 9]. However, people may be reluctant to use CHX mouthwash as prescribed as it can cause tooth staining [10, 11]. As staining levels are associated with plaque occurrence, one way to help counteract stain is by minimising plaque build-up through, for instance, the use of NaHCO_3_-containing dentifrice, which can help physically remove plaque biofilm [13, 23].

In this study, stain accumulation at 6 weeks was higher than had been seen at baseline, suggesting that indeed CHX use led to increased levels of staining. While the 67 % NaHCO_3_ dentifrice group showed a numerically lower level of stain compared to the Control dentifrice group, the difference in Overall MLSI (IxA) (a combined measure of maxillary and mandibular facial and mandibular lingual staining) was not significant at either 3 or 6 weeks. The Overall Facial MLSI was statistically significantly different at Week 6, favouring the 67 % NaHCO_3_ dentifrice. This was the only measure to exclude the lingual surface, which is of interest as the facial surfaces are known to be easier to clean than the lingual ones.

To examine factors that may have led to a finding of non-significance overall, the study results were broken down by site in a post-hoc analysis. For Site 1 there were significant differences favouring the 67 % NaHCO_3_ dentifrice at both weeks and for all areas including overall MLSI; this was not shown for Site 2. Examiner variations were ruled out as the same examiner was used at both sites and the same hygienist carried out the majority of the prophylaxes at both sites. The randomisation process and storage conditions of the study supplies were also checked and no issues found. There were differences in the clinical suite lighting: Site 1 used a 5000 K bulb, Site 2 a 3550 K bulb, but this was not thought to affect assessments as there were similarities in baseline data between study sites. Another factor could be that there was a difference in the consumption of stain-causing drinks by site; however, the fact that baseline staining and Week 6 control dentifrice values were very similar may not support this postulate.

A factor of interest was that there was a difference in water hardness with Site 1 (Manchester) classified as ‘very soft’ (average 9.3 milligrams/litre calcium [mg/l Ca]) [24] and Site 2 (Maldon) as ‘hard to very hard’ (range 98–120 mg/l Ca) [25]. It was queried as to whether or not this could have affected the results. An in vitro study showing that staining intensity with a combination of tea and CHX mouthwash was greater with hard water [26] would mean that this current study should have had higher Week 6 staining levels with the Control dentifrice at Site 2, which was not the case; hence, any differences could be due to potential interaction factors between water hardness and the effectiveness of NaHCO_3_ as opposed to being due to interactions with the CHX mouthwash.

## Conclusion

In summary, no significant difference in stain levels was found between a dentifrice containing NaHCO_3_ and one without this ingredient when using a mouthwash containing 0.2 % CHX. Differences were seen in facial surface staining after 6 weeks, suggesting effectiveness of NaHCO_3_ dentifrice on more accessible surfaces. As differences were seen at different sites, further investigation may be warranted.

## Abbreviations

AE: Adverse events
ANCOVA: Analysis of covariance
B: Body
BI: Saxton & Van der Ouderra bleeding index
BL: Baseline
Ca: Calcium
CHX: Chlorhexidine digluconate
CI: Confidence interval
D: Distal
Diff: Difference
G: Gingival
GSKCH: GSK Consumer Healthcare
ITT: Intent-to-treat
IxA: Intensity multiplied by area
M: Mesial
Man-F: Mandibular-facial
Man-L: Mandibular-lingual
Max-F: Maxillary-facial
Mg/l: Milligrams/litre
MLSI: Modified Lobene stain index
MW: Mouthwash
n (%): Number (percent) of subjects with at least one AE
n: Number
nAE: Number of adverse events
NaF: Sodium fluoride
NaHCO_3_: Sodium bicarbonate
OST: Oral soft tissue
PP: Per protocol
PROPHY: Prophylaxis
RDA: Relative dentin abrasivity
SD: Standard deviation

## References

[1] Petersen PE, Bourgeois D, Ogawa H, Estupinan-Day S, Ndiaye C (2005). The global burden of oral diseases and risks to oral health. Bull World Health Organ.

[2] Haps S, Slot DE, Berchier CE, Van der Weijden GA (2008). The effect of cetylpyridinium chloride-containing mouth rinses as adjuncts to toothbrushing on plaque and parameters of gingival inflammation: a systematic review. Int J Dent Hyg.

[3] Varoni E, Tarce M, Lodi G, Carrassi A (2012). Chlorhexidine (CHX) in dentistry: state of the art. Minerva Stomatol.

[4] Emilson CG (1977). Susceptibility of various microorganisms to chlorhexidine. Scand J Dent Res.

[5] Eick S, Goltz S, Nietzsche S, Jentsch H, Pfister W (2011). Efficacy of chlorhexidine digluconate-containing formulations and other mouthrinses against periodontopathogenic microorganisms. Quintessence Int.

[6] Bonesvoll P (1977). Oral pharmacology of chlorhexidine. J Clin Periodontol.

[7] Löe H, Schiøtt CR (1970). The effect of mouthrinses and topical application of chlorhexidine on the development of dental plaque and gingivitis in man. J Periodontal Res.

[8] Segreto VA, Collins EM, Beiswanger BB, de la Rosa RL, Isaacs RL, Lang NP (1986). A comparison of mouthrinses containing two concentrations of chlorhexidine. J Periodontal Res.

[9] Gunsolley JC (2010). Clinical efficacy of antimicrobial mouthrinses. J Dent.

[10] Van Strydonck DA, Slot DE, Van der Velden U, Van der Weijden F (2012). Effect of a chlorhexidine mouthrinse on plaque, gingival inflammation and staining in gingivitis patients: a systematic review. J Clin Periodontol.

[11] Flötra L, Gjermo P, Rölla G, Waerhaug J (1971). Side effects of chlorhexidine mouth washes. Scand J Dent Res.

[12] Zanatta FB, Antoniazzi RP, Rösing CK (2010). Staining and calculus formation after 0.12 % chlorhexidine rinses in plaque-free and plaque covered surfaces: a randomized trial. J Appl Oral Sci.

[13] Putt MS, Milleman KR, Ghassemi A (2008). Enhancement of plaque removal efficacy by tooth brushing with baking soda dentifrices: results of five clinical studies. J Clin Dent.

[14] Schemehorn BR, Moor MH, Putt MS (2011). Abrasion, polishing and stain removal characteristics of various commercial dentifrices in vitro. J Clin Dent.

[15] Lehne RK, Winston AE (1983). Abrasivity of sodium bicarbonate. Clin Prev Dent.

[16] GSK Clinical Study Register. http://www.gsk-clinicalstudyregister.com/study/202182?study_ids=RH01913#rs. Accessed 03 Aug 2016.

[17] World Medical Association Declaration of Helskinki. Ethical Principles for Medical Research Involving Human Subjects. 59th General Assembly, Seoul 2008. http://www.wma.net/en/30publications/10policies/b3/17c.pdf. Accessed 17 Aug 2016.

[18] Saxton CA, Van der Ouderra FJ (1989). The effect of a dentifrice containing zinc citrate and triclosan on developing gingivitis. J Periodontal Res.

[19] Lobene RR (1968). Effects of dentifrices on tooth stains with controlled brushing. J Am Dent Assoc.

[20] MacPherson LM, Stephen K, Joiner A, Schafer F, Huntington E (2000). Comparison of a conventional and modified tooth stain index. J Clin Periodontol.

[21] Yankell SL, Emling RC, Prencipe M, Rustogi K, Volpe AR (1994). Clinical study to assess the stain removal efficacy of two tartar control dentifrices and a low abrasive dentifrice. J Clin Dent.

[22] Jorgensen E, Pedersen AR. How to obtain those nasty standard errors from transformed data – and why they should not be used. Internal report by Aarhus Universitet, Det Jordbrugsvidenskabelige Fakultet, 1997. p20. http://citeseerx.ist.psu.edu/viewdoc/download?doi=10.1.1.47.9023&rep=rep1&type=pdf. Accessed 17 Aug 2016.

[23] Ghassemi A, Vorwerk LM, Hooper WJ, Putt MS, Milleman KR (2008). A four-week clinical study to evaluate and compare the effectiveness of a baking soda dentifrice and an antimicrobial dentifrice in reducing plaque. J Clin Dent.

[24] United Utilities. My drinking water quality. http://www.unitedutilities.com/waterquality.aspx?postcode=M9+8ES. Accessed 03 Aug 2016.

[25] Essex & Suffolk Water. Water hardness. https://www.eswater.co.uk/_assets/documents/3122_ESW_Water_hardness.pdf. Accessed 03 Aug 2016.

[26] Sarembe S, Kiesow A, Petzold M (2010). Investigations of Dental Staining Considering the Water Hardness in vitro.

